# Supplementary material for: Exploring support for medicalized female genital mutilation/cutting: A study on migrant women living in Italy
Source: PLoS One. 2025 May 7;20(5):e0322774. doi: 10.1371/journal.pone.0322774 (PMC12057872; doi:10.1371/journal.pone.0322774)
Supplement: S1 Appendix — Multinomial probit choice models. Coefficients and robust standard errors from Model 1a and Model 2a. (DOCX) [file pone.0322774.s001.docx]

| Table A1. Multinomial probit choice models. Coefficients and robust standard errors from Model 1a and Model 2a. | | | | | | |
| --- | --- | --- | --- | --- | --- | --- |
|  | Model 1: full sample | | | Model 2: only cut women | | |
| alternative-specific variables | Coefficient | Robust standard error | P>\|z\| | Coefficient | Robust standard error | P>\|z\| |
| Perceived benefits of FGM/C: Cleanliness/hygiene (Yes; No, reference category) | 1.247 | 0.463 | 0.007 | 1.859 | 0.379 | <0.001 |
| Perceived benefits of FGM/C: Social acceptance (Yes; No, reference category) | -0.679 | 0.495 | 0.170 | -0.720 | 0.731 | 0.325 |
| Perceived benefits of FGM/C: Better marriage prospects (Yes; No, reference category) | 1.632 | 0.340 | <0.001 | 1.918 | 0.409 | <0.001 |
| Perceived benefits of FGM/C: Preserve virginity/Prevent premarital sex (Yes; No, reference category) | 1.328 | 0.372 | <0.001 | 1.292 | 0.422 | 0.002 |
| Perceived benefits of FGM/C: Preserve cultural traditions of parents/ancestors (Yes; No, reference category) | 0.718 | 0.274 | 0.009 | 0.800 | 0.424 | 0.059 |
| Perceived benefits of FGM/C: To instil discipline and traditional cultural values (Yes; No, reference category) | 1.280 | 0.333 | <0.001 | 1.526 | 0.429 | <0.001 |
| Perceived benefits of FGM/C: Religious approval (Yes; No, reference category) | 2.126 | 0.705 | 0.003 | 2.545 | 0.742 | 0.001 |
| Perceived benefits of FGM/C: More pleasure for men (Yes; No, reference category) | 1.003 | 0.623 | 0.107 | 1.093 | 0.709 | 0.123 |
| *women's specific variables - Choice: Support for FGM/C under the condition of medicalisation vs Unconditional Support for FGM/C* |  |  |  |  |  |  |
| Age at migration | -0.103 | 0.042 | 0.014 | -0.067 | 0.044 | 0.128 |
| Age at the survey | 0.115 | 0.040 | 0.004 | 0.114 | 0.043 | 0.008 |
| Higher level of achieved formal education: secondary (ref. None or Primary) | 0.732 | 0.326 | 0.025 | 1.025 | 0.279 | <0.001 |
| Higher level of achieved formal education: tertiary (ref. None or Primary) | 1.364 | 0.468 | 0.004 | 1.540 | 0.497 | 0.002 |
| The woman is active in the labour market: Yes (ref. No, reference) | 0.035 | 0.440 | 0.937 | 0.741 | 0.391 | 0.058 |
| Family status: In a relationship living apart (ref. Single/separated/widowed) | 0.482 | 0.561 | 0.390 | 0.297 | 0.549 | 0.589 |
| Family status: In a relationship living together (ref. Single/separated/widowed) | 0.238 | 0.546 | 0.664 | 1.107 | 0.501 | 0.027 |
| The woman is married to an Italian native: Yes (ref. No, reference) | -1.775 | 0.491 | 0.000 | -2.869 | 0.337 | <0.001 |
| The woman is cut: Yes (ref. No, reference) | -0.848 | 0.386 | 0.028 |  |  |  |
| The woman regularly returns to the country of origin: Yes (ref. No, reference) | 0.270 | 0.276 | 0.327 | 0.232 | 0.253 | 0.359 |
| Number of female daughters | 0.013 | 0.198 | 0.947 | 0.019 | 0.181 | 0.915 |
| Prevalence of medicalisation in the country of origin | -0.011 | 0.015 | 0.450 | -0.016 | 0.019 | 0.380 |
| Constant | 1.272 | 0.771 | 0.099 | -1.110 | 0.782 | 0.156 |
| *women's specific variables - Choice: No support vs Unconditional Support for FGM/C* |  |  |  |  |  |  |
| Age at migration | -0.037 | 0.027 | 0.177 | -0.031 | 0.027 | 0.242 |
| Age at the survey | 0.072 | 0.026 | 0.006 | 0.069 | 0.025 | 0.006 |
| Higher level of achieved formal education: secondary (ref. None or Primary) | 0.631 | 0.160 | <0.001 | 0.674 | 0.158 | <0.001 |
| Higher level of achieved formal education: tertiary (ref. None or Primary) | 0.788 | 0.261 | 0.002 | 0.705 | 0.262 | 0.007 |
| The women is active in the labour market: Yes (ref. No, reference) | -0.129 | 0.208 | 0.534 | 0.005 | 0.213 | 0.982 |
| Family status: In a relationship living apart (ref. Single/separated/widowed) | 1.107 | 0.191 | <0.001 | 0.988 | 0.160 | <0.001 |
| Family status: In a relationship living together (ref. Single/separated/widowed) | 0.483 | 0.291 | 0.097 | 0.634 | 0.314 | 0.043 |
| The woman is married to an Italian native: Yes (ref. No, reference) | -1.094 | 0.803 | 0.173 | -1.381 | 0.886 | 0.119 |
| The woman is cut: Yes (ref. No, reference) | 0.737 | 0.504 | 0.143 |  |  |  |
| The woman regularly returns to the country of origin: Yes (ref. No, reference) | -0.029 | 0.239 | 0.902 | -0.089 | 0.208 | 0.669 |
| Number of female daughters | -0.301 | 0.104 | 0.004 | -0.291 | 0.102 | 0.004 |
| Prevalence of medicalisation in the country of origin | 0.022 | 0.009 | 0.015 | 0.017 | 0.009 | 0.060 |
| Constant | -3.005 | 0.514 | <0.001 | -2.330 | 0.532 | <0.001 |
| Covariance factor loadings | 0.497 | 0.231 | 0.031 | 0.396 | 0.233 | 0.089 |
| AIC | 776.398 |  |  | 621.241 |  |  |
