# Supplementary material for: Exploring support for medicalized female genital mutilation/cutting: A study on migrant women living in Italy
Source: PLoS One. 2025 May 7;20(5):e0322774. doi: 10.1371/journal.pone.0322774 (PMC12057872; doi:10.1371/journal.pone.0322774)
Supplement: S2 Appendix — Multinomial probit choice models. Coefficients and robust standard errors from Model 1b and Model 2b. (DOCX) [file pone.0322774.s002.docx]

Table A2. Multinomial probit choice models. Coefficients and robust standard errors from Model 1b and Model 2b.

|  | Model 1: full sample | | | Model 2: only cut women | | |
| --- | --- | --- | --- | --- | --- | --- |
| alternative-specific variables | Coefficient | Robust standard error | P>\|z\| | Coefficient | Robust standard error | P>\|z\| |
| Perceived benefits of FGM/C: Cleanliness/hygiene (Yes; No, reference category) | 1.187 | 0.428 | 0.006 | 1.677 | 0.339 | <0.001 |
| Perceived benefits of FGM/C: Social acceptance (Yes; No, reference category) | -0.595 | 0.491 | 0.226 | -0.621 | 0.681 | 0.362 |
| Perceived benefits of FGM/C: Better marriage prospects (Yes; No, reference category) | 1.583 | 0.362 | 0.000 | 1.746 | 0.362 | <0.001 |
| Perceived benefits of FGM/C: Preserve virginity/Prevent premarital sex (Yes; No, reference category) | 1.238 | 0.368 | 0.001 | 1.141 | 0.407 | 0.005 |
| Perceived benefits of FGM/C: Preserve cultural traditions of parents/ancestors (Yes; No, reference category) | 0.695 | 0.282 | 0.014 | 0.724 | 0.402 | 0.072 |
| Perceived benefits of FGM/C: To instil discipline and traditional cultural values (Yes; No, reference category) | 1.284 | 0.324 | 0.000 | 1.415 | 0.407 | 0.001 |
| Perceived benefits of FGM/C: Religious approval (Yes; No, reference category) | 2.046 | 0.742 | 0.006 | 2.304 | 0.748 | 0.002 |
| Perceived benefits of FGM/C: More pleasure for men (Yes; No, reference category) | 0.943 | 0.601 | 0.117 | 0.988 | 0.640 | 0.123 |
| *women's specific variables - Choice: Support for FGM/C under the condition of medicalisation vs No support* |  |  |  |  |  |  |
| Age at migration | 0.061 | 0.013 | <0.001 | 0.031 | 0.013 | 0.015 |
| Age at the survey | -0.035 | 0.007 | <0.001 | -0.033 | 0.010 | 0.001 |
| Higher level of achieved formal education: secondary (ref. None or Primary) | 0.036 | 0.272 | 0.895 | -0.136 | 0.230 | 0.556 |
| Higher level of achieved formal education: tertiary (ref. None or Primary) | -0.403 | 0.282 | 0.153 | -0.590 | 0.296 | 0.047 |
| The woman is active in the labour market: Yes (ref. No, reference) | -0.169 | 0.253 | 0.505 | -0.648 | 0.297 | 0.029 |
| Family status: In a relationship living apart (ref. Single/separated/widowed) | 0.740 | 0.566 | 0.192 | 0.812 | 0.585 | 0.165 |
| Family status: In a relationship living together (ref. Single/separated/widowed) | 0.292 | 0.560 | 0.603 | -0.273 | 0.565 | 0.629 |
| The woman is married to an Italian native: Yes (ref. No, reference) | 0.671 | 0.607 | 0.269 | 1.222 | 0.648 | 0.059 |
| The woman is cut: Yes (ref. No, reference) | 1.482 | 0.468 | 0.002 |  |  |  |
| The woman regularly returns to the country of origin: Yes (ref. No, reference) | -0.299 | 0.228 | 0.191 | -0.329 | 0.235 | 0.160 |
| Number of female daughters | -0.347 | 0.134 | 0.009 | -0.339 | 0.132 | 0.010 |
| Prevalence of medicalisation in the country of origin | 0.034 | 0.006 | <0.001 | 0.032 | 0.009 | <0.001 |
| Constant | -4.434 | 0.913 | <0.001 | -1.687 | 0.854 | 0.048 |
| *women's specific variables - Choice: Unconditional Support for FGM/C vs No support* |  |  |  |  |  |  |
| Age at migration | 0.106 | 0.039 | 0.006 | 0.068 | 0.043 | 0.114 |
| Age at the survey | -0.122 | 0.036 | 0.001 | -0.116 | 0.040 | 0.003 |
| Higher level of achieved formal education: secondary (ref. None or Primary) | -0.824 | 0.247 | 0.001 | -1.071 | 0.209 | <0.001 |
| Higher level of achieved formal education: tertiary (ref. None or Primary) | -1.480 | 0.364 | <0.001 | -1.550 | 0.365 | <0.001 |
| The women is active in the labour market: Yes (ref. No, reference) | -0.006 | 0.470 | 0.990 | -0.670 | 0.424 | 0.114 |
| Family status: In a relationship living apart (ref. Single/separated/widowed) | -0.623 | 0.563 | 0.269 | -0.436 | 0.510 | 0.392 |
| Family status: In a relationship living together (ref. Single/separated/widowed) | -0.286 | 0.533 | 0.592 | -1.098 | 0.418 | 0.009 |
| The woman is married to an Italian native: Yes (ref. No, reference) | 1.831 | 0.542 | 0.001 | 2.862 | 0.420 | <0.001 |
| The woman is cut: Yes (ref. No, reference) | 0.888 | 0.394 | 0.024 |  |  |  |
| The woman regularly returns to the country of origin: Yes (ref. No, reference) | -0.249 | 0.299 | 0.405 | -0.168 | 0.241 | 0.484 |
| Number of female daughters | 0.018 | 0.212 | 0.932 | 0.031 | 0.189 | 0.868 |
| Prevalence of medicalisation in the country of origin | 0.009 | 0.015 | 0.550 | 0.013 | 0.017 | 0.460 |
| Constant | -1.176 | 0.917 | 0.200 | 1.155 | 0.732 | 0.114 |
| Covariance factor loadings | 1.084 | 0.277 | <0.001 | 0.939 | 0.254 | <0.001 |
| AIC | 778.072 |  |  | 623.140 |  |  |
