# Supplementary material for: Exploring support for medicalized female genital mutilation/cutting: A study on migrant women living in Italy
Source: PLoS One. 2025 May 7;20(5):e0322774. doi: 10.1371/journal.pone.0322774 (PMC12057872; doi:10.1371/journal.pone.0322774)
Supplement: Attachment — Submitted filename: Answer to reviewers.docx [file pone.0322774.s004.docx]

Dear Editor and reviewers,

Thank you for giving us the opportunity to carry out the minor revisions requested for publication in PLOS One.

We met the suggestions when possible and details are reported below (in red) as point by-point answers to observations.

Kind Regards

The Authors

**Comments to the Author**

1. Is the manuscript technically sound, and do the data support the conclusions?

The manuscript must describe a technically sound piece of scientific research with data that supports the conclusions. Experiments must have been conducted rigorously, with appropriate controls, replication, and sample sizes. The conclusions must be drawn appropriately based on the data presented.

Reviewer #1: Yes

Reviewer #2: Partly

Reviewer #3: Yes

2. Has the statistical analysis been performed appropriately and rigorously?

Reviewer #1: Yes

Reviewer #2: Yes

Reviewer #3: Yes

3. Have the authors made all data underlying the findings in their manuscript fully available?

The [PLOS Data policy](http://www.plosone.org/static/policies.action#sharing) requires authors to make all data underlying the findings described in their manuscript fully available without restriction, with rare exception (please refer to the Data Availability Statement in the manuscript PDF file). The data should be provided as part of the manuscript or its supporting information, or deposited to a public repository. For example, in addition to summary statistics, the data points behind means, medians and variance measures should be available. If there are restrictions on publicly sharing data—e.g. participant privacy or use of data from a third party—those must be specified.

Reviewer #1: Yes

Reviewer #2: Yes

Reviewer #3: No

4. Is the manuscript presented in an intelligible fashion and written in standard English?

PLOS ONE does not copyedit accepted manuscripts, so the language in submitted articles must be clear, correct, and unambiguous. Any typographical or grammatical errors should be corrected at revision, so please note any specific errors here.

Reviewer #1: Yes

Reviewer #2: Yes

Reviewer #3: Yes

5. Review Comments to the Author

Please use the space provided to explain your answers to the questions above. You may also include additional comments for the author, including concerns about dual publication, research ethics, or publication ethics. (Please upload your review as an attachment if it exceeds 20,000 characters)

Reviewer #1: The authors have conducted the research in a rigorous manner. The data depicted in the findings section supports the final conclusion and recommendations. The findings will definitely add to the knowledge base of the subject area. The authors have good command of English language which makes it easy to read and understand.

Reviewer #2: It would be appropriate to use references that are not more than 5 years to make the study more current.

Thank you for the suggestions. We have updated older references whenever possible. However, in some instances, we were unable to replace the cited references with more recent findings. As a result, we have retained these references in the text as they are still relevant for framing the study.

under the Methodology section, please organize it well under Methods and Materials and let there be clarity with the step by step ways that was used to achieve the results. otherwise replicability is not possible. You are trying to analyze an already qualitative study quantitatively and that should come with a step by step clarity.
Please add the qualitative data as a supplementary file or add the link where that can be assessed.

We hope we have clearly understood the reviewer's request. We have renamed the section to "Methods and Materials". We also specified that the selected model required us to reshape the original data into long form, and no other steps were carried out on the original data for the analysis. The rest of the analysis was carried out in Stata using cm and cmmprobit commands along with standard post-estimation commands.

We also want to clarify here that our data are fully quantitative, and no qualitative data (mentioned above) were produced at any stage. Data for the study comes from the 2016 national survey on Female Genital Mutilation conducted in Italy as a part of the Daphne project FGM-Prev, which is a quantitative study. The study received ethical clearance from the ethical committee of the University of Milan-Bicocca. The survey was conducted from June to December 2016, covering a representative sample of 1,378 women aged 18+ living in Italy and born in Nigeria, Egypt, Eritrea, Senegal, Burkina Faso, Somalia and the Ivory Coast. Details regarding the data have been updated as per the Journal's checklist. We are open to sharing the codes; however, as explained in the submission, the data are not publicly available.

Reviewer #3: A research study was conducted which explores how migrant women from countries where FGM/C is practiced, living in Italy, view the medicalization of FGM/C and the traits of those who endorse this practice. The findings suggest that compared to women who unconditionally support the practice, those who advocate for medicalisation tend to have higher levels of education, be older, be in relationships, and come from countries where FGM/C is more frequently medicalised. Perceived advantages associated with greater endorsement of FGM/C medicalisation include religious sanction, improved marriage opportunities, hygiene considerations, and adherence to traditional cultural norms.

Minor revisions:
1- Table 2: Include the standard deviations that correspond to the means provided in this table.

Added

2- Table 6: Indicate the meaning of the triple asterisks.

Added

6. PLOS authors have the option to publish the peer review history of their article ([what does this mean?](https://journals.plos.org/plosone/s/editorial-and-peer-review-process#loc-peer-review-history)). If published, this will include your full peer review and any attached files.


If you choose “no”, your identity will remain anonymous but your review may still be made public.


**Do you want your identity to be public for this peer review?** For information about this choice, including consent withdrawal, please see our [Privacy Policy](https://www.plos.org/privacy-policy).

Reviewer #1: No

Reviewer #2: No

Reviewer #3: No

[NOTE: If reviewer comments were submitted as an attachment file, they will be attached to this email and accessible via the submission site. Please log into your account, locate the manuscript record, and check for the action link "View Attachments". If this link does not appear, there are no attachment files.]

While revising your submission, please upload your figure files to the Preflight Analysis and Conversion Engine (PACE) digital diagnostic tool, <https://pacev2.apexcovantage.com/>. PACE helps ensure that figures meet PLOS requirements. To use PACE, you must first register as a user. Registration is free. Then, login and navigate to the UPLOAD tab, where you will find detailed instructions on how to use the tool. If you encounter any issues or have any questions when using PACE, please email PLOS at <a href="mailto:figures@plos.org">figures@plos.org. Please note that Supporting Information files do not need this step.
