# Supplementary material for: Exploring support for medicalized female genital mutilation/cutting: A study on migrant women living in Italy
Source: PLoS One. 2025 May 7;20(5):e0322774. doi: 10.1371/journal.pone.0322774 (PMC12057872; doi:10.1371/journal.pone.0322774)
Supplement: Attachment — Submitted filename: answer_to_reviewers_auresp_2.docx [file pone.0322774.s005.docx]

Reviewer #1: The authors work is carried out in a rigorous manner. The conclusions and recommendations are well supported by the findings of the study. This work has a great potential to add to the scientific knowledge in this field.

Thank you

Reviewer #2: Line 99: 'At least to our knowledge.' Please delete this phrase and use a more appropriate scholarly phrase.

Done
line 121-276: Please, fuse/merge the important points with the Introduction, or better separate it as as a review paper.

We separated it as subsection in the section “theoretical background”

Instead of 'We' and 'Our' use this study. For example, line 334 can read 'The dependent variables was considered based on this question':
We rephased as suggested

Please engage an English editor.

Done. Please consider that version with track changes refers to the paper before proofreading. Differences with the clean version are only due to language improvement.

Lines 347-373; please put it in a table.

Done, table have been renamed accordingly

line 506-'Our" knowledge' please delete

Done
311-320: Please attach as a supplementary

This part details the methodology and cannot be shown only in the supplementary materials. Also, reviewer 5 asks for more details instead.

Reviewer #3: Minor revisions:
1- Table 2: For data summarized as percentages, add the corresponding sample size.

Added
2- Table 6: Replace the "***" in the table with "<.001" which is less confusing since all p-values are <.001.

Done
3- Several tables: P-values never equal zero. Express small p-values as <.001.

Done, also in the appendix

Reviewer #4: I would like to congratulate the authors for directing attention to the voices of small but brave respondents who expressed their support for medicalisation of FGM/C. No doubt this has a lot to offer in further exploration of the under-explored motivations for this support. This study though completed in Italy, is an eye opener for other destination countries for migrants such as Australia, UK and USA to invest in similar revisit of existing data or conduct new research.

It was a pleasure reviewing this revised version of the manuscript.

Best wishes.

Thank you

Reviewer #5: I would like to congratulate the authors, this is truly fascinating paper based on a unique data set which significantly advances our understanding of attitudes to medicalized FGMC among migrant women in Italy. This paper will certainly be well cited, as it provides unique and important details on attitudes and needs of women who have migrated to Europe from high FGMC contexts, but also provides some insights into how attitudes may change overtime, towards medicalisation.

In can confirm that the authors have already made all the revisions required by reviewers 1 2, and 3, In addition, I would like to offer the authors’ the following comments and suggestions.

1) The authors report that FGMC support maybe under-reported in their data. In the discussion, the implications of mis-reporting for their main findings should be outlined. Specifically, I’m concerned that mis-reporting may not be equal across their sample, and that there could be a tendency for both under-reporting and even over-reporting among different sub-groups. For example, highly educated women may be more likely to mis-report in some way. A previous study that used both indirect and direct methods found that the most highly educated people are more likely to give socially desirable answers, under-reporting their “true” preferences for FGMC in self-reports (Gibson et al 2018).

Thank you for this crucial observation. As in most FGM/C surveys, it is not possible to control for misreport of FGM/C status or support (the cited text is a notable exception). We add this reference to the discussion.

Is indicating that you prefer medicalized FGMC (which as you say sound more hygienic, lower health risk) a more socially desirable answer in the context of a survey undertaken in Italy? Or can we assume that all mis-reporting is equal across the women included in the survey – I’m not sure it can as its quite a diverse sample. The author should add this to the limitation section of the discussion, particularly re: the potential for educated people to give socially desirable responses to questions on FGMC support which has been shown before.

Thank you for this crucial observation. As in most FGM/C surveys, it is not possible to control for misreport of FGM/C status or support (the cited text is a notable exception). We add this reference to the discussion. However we do not have solid ground to affirm that social desirability may lead women to declare support for medicalization while they could more easily declare they are against the practice. While reporting support for abandonment may disclose hidden support for the practice, we think that medicalization may be less biased as a choice.

This is what we added to the text “these data share most of the expected limitations of surveys on hard-to-reach populations [51] and surveys based on self-reported data on FGM/C status that may be affected by under-reporting of the experience of FGM or for the support of its continuation driven by social desirability [53, 54]. While support for medicalised FGM/C may be as affected by underreporting as unbiased support for FGM/C, there is the possibility that some women may declare a support conditioned to medicalisation due social desirability, while they unconditionally support the practice. However, women particularly oriented towards socially desirable answers are expected to opt for disclosing their support rather than conditioning it to medicalisation.”

2) Also in the limitations section of the discussion. The authors may wish to explicitly discuss the implications of removing women who indicated they were “unsure” or refused to answer the survey. The sample may also be biased here. They should also indicate in the Measures section exactly how many women were excluded from the analyses.

Thank you for your request, which allowed us to disentangle the issue. 57 women are excluded as they declared they had never heard of FGM/C (4.1%). For this reason, they were not asked about their support or intention. Among those who have heard of FGM/C 16 (1.2%) refused to answer and 57 (4.1%) were unsure. Those who are unsure or refuse to answer are excluded because we cannot attribute to their choice any FGM/C-choice related characteristic. However, we lose for this reason only 6.9% of the women aware of FGM/C.

3) Methods/ Discrete choice framework – further details of the survey and the DCE framework would be useful in the methods section. How were the items selected, and how were they presented to respondents? A real benefit of the DCE in measuring preferences, is that when designed well it can it mitigates against strategic biases.

The choice derives from the answer to the item “Do you think that this practice [FGM/C] should continue?”. Possible answers were “No”, “Yes”, “Yes but only if carried out in a hospital”, “Unsure”, and “Refuses to answer”. Women who agreed with the third option were therefore considered favourable to FGM/C conditionally on medicalisation.

This is explained in the Method section. So choices are framed accordingly. This strategy helps avoiding social desirability bias as it is not framed in terms of intention on the body of their daughter but in terms of an opinion.

4) “Regression towards unbiased favour” p23. I think the authors are making reference to the expectation that FGMC changes occur in one direction – from traditional cutting methods to medicalization to full abandonment. This is an interesting point and I wonder if this is theoretical or has actually been empirically tested? A bit more explanation would be helpful here. My understanding from the qualitative/anthropological literature is that FGMC may emerge 'de novo' or re-emerge under the right circumstances, so that we can’t be certain on of a simple pathways to abandonment.

This is an interesting observation. There is no evidence in the literature on countries of origin or among migrants of a return or new emergence of the practice where it is not present or has been abandoned or of an abandonment of medicalization for unmedicalized practice. Reasons are also discussed in the theoretical section in terms of the relationship with the ideas of safety and modernity. However, we slightly changed the text by adding some references. We also explain that a reason not to expect change from support to medicalization to unbiased medicalisation is due to the profile of women who are more educated and, therefore, are more likely to consider health-related risks.

5) Small points:
a. I suggest moving the bullet-point list of explanatory variables on p13 into a table for ease of reading and improved structure.

Done, also in line with suggestion from reviewer 1

b. P15, end of first paragraph. There is a mention of model which controls for country of origin, but is not presented as the authors say they use a proxy for a “better fit”. Perhaps in the interests of transparency, this could be added to the Supporting Information.

We deleted this sentence, not to add too many materials that are not really useful for the readers. By using the country of origin instead of the prevalence, we lose information about the prevalence of medicalization that is much more informative and relevant for the interpretation of results while having a worst fit.

Gibson MA, Gurmu E, Cobo B, Rueda MM, Scott IM (2018) Indirect questioning method reveals hidden support for female genital cutting in South Central Ethiopia. PLOS ONE 13(5): e0193985. <https://doi.org/10.1371/journal.pone.0193985>

7. PLOS authors have the option to publish the peer review history of their article ([what does this mean?](https://track.editorialmanager.com/CL0/https:%2F%2Fjournals.plos.org%2Fplosone%2Fs%2Feditorial-and-peer-review-process%23loc-peer-review-history/1/010f019394ed269c-f3e7fd9d-e043-4d6e-949d-4fa1817a000c-000000/Bsr3mUGGaZrmNhHOgn848nY7NVpZK2qc7BxqF4Q0yjE=187)). If published, this will include your full peer review and any attached files.


If you choose “no”, your identity will remain anonymous but your review may still be made public.


**Do you want your identity to be public for this peer review?** For information about this choice, including consent withdrawal, please see our [Privacy Policy](https://track.editorialmanager.com/CL0/https:%2F%2Fwww.plos.org%2Fprivacy-policy/1/010f019394ed269c-f3e7fd9d-e043-4d6e-949d-4fa1817a000c-000000/igauW9x_2ynpESynnV3e5PsKoaOIIlahjSNzYhfz494=187).

Reviewer #1: **Yes:** Jane W. Kabo

Reviewer #2: No

Reviewer #3: No

Reviewer #4: **Yes:** Associate Professor Olayide Ogunsiji

Reviewer #5: **Yes:** Mhairi Gibson
