# Supplementary material for: Exploring support for medicalized female genital mutilation/cutting: A study on migrant women living in Italy
Source: PLoS One. 2025 May 7;20(5):e0322774. doi: 10.1371/journal.pone.0322774 (PMC12057872; doi:10.1371/journal.pone.0322774)
Supplement: Attachment — Submitted filename: Answer_to_reviewers_auresp_3.docx [file pone.0322774.s006.docx]

**Comments to the Author**

Review Comments to the Author

Please use the space provided to explain your answers to the questions above. You may also include additional comments for the author, including concerns about dual publication, research ethics, or publication ethics. (Please upload your review as an attachment if it exceeds 20,000 characters)

Reviewer #3: Minor revisions:
1- Table 2: For data summarized as percentages, add the corresponding sample size.
2- Table 6: Replace the "***" in the table with "<.001" which is less confusing since all
p-values are <.001.
3- Several tables: P-values never equal zero. Express small p-values as <.001.

All these comments had already been addressed in the previous round of revision. No additional changes needed to be made.

Please note that Table 2 was renumbered into Table 3 in the previous revision round.

Reviewer #5: Thank you for addressing the questions and comments constructively. It is a very interesting and worthwhile analyses. In general the manuscript reads well, however I note that some of the additional/clarifying text which has been added to R2 version needs some simple editing/rephrasing to improve clarity.
Line 505 - if I understand correctly, "unbiased support for FGMC" should be "unconditional support for FGMC"?

We changed the term “unbiased” into “unconditional” support for better clarity

Line 507 - needs an extra clause or sentence to explain your argument. You express it clearly and concisely in your response to reviewers document - that it is your expectation that the most likely scenario is that women declare they are against the practice or similar. The text inserted in the manuscript and that pasted into the response to reviewer document are slightly different, so it might be worth checking this section carefully.

Thank you. Building on the answer provided in the rebuttal letter you mentioned, we added this phrase in the text: “There is no solid ground to affirm that social desirability may lead women to declare support for medicalization while they could more easily declare they are against the practice”.

Also, the text inserted in the answer differed slightly from the paper because the response letter was prepared before the text was proofread, as requested by another reviewer. Sorry about this.

Line 521 - the statement "while the second group needs to be addressed, considering medicalization an an evolved form of FGMC" is unclear.

We changed into: “The first group might be complicit in combating the practice within their communities, while the second group has to be addressed, with special attention also to women seeking medicalized FGM/C for their children”.

Line 522-3, ditto. Maybe replace "which may instead be fuelled by" with "in"

Done

Line 525. "one does not expect a regression to an unbiased favour" needs rewording to improve clarity. I expect unbiased favour could be replaced with unconditional support. I would avoid first person terms like "one", and also the term "regression" for different reasons.

We changed into: “Finally, a return toward unconditional support for FGM/C from women who are in favor of FGM/C, as long as it is medicalized, is not expected. However, because of their profile, medicalization cannot necessarily be considered an intermediate step toward abandonment”.

For the same reasons, we changed the term “evolve” into “move” in another section of the text.

7. PLOS authors have the option to publish the peer review history of their article ([what does this mean?](https://track.editorialmanager.com/CL0/https:%2F%2Fjournals.plos.org%2Fplosone%2Fs%2Feditorial-and-peer-review-process%23loc-peer-review-history/1/010f01958cf4de52-5676ca4b-8b13-407b-bb18-b076d11737c8-000000/iDzfTKnts9rjnsM0pNy8yaWF2BfxB0taJd4e8dbTu24=202)). If published, this will include your full peer review and any attached files.


If you choose “no”, your identity will remain anonymous but your review may still be made public.


**Do you want your identity to be public for this peer review?** For information about this choice, including consent withdrawal, please see our [Privacy Policy](https://track.editorialmanager.com/CL0/https:%2F%2Fwww.plos.org%2Fprivacy-policy/1/010f01958cf4de52-5676ca4b-8b13-407b-bb18-b076d11737c8-000000/YGVvXu5-syYODO8wAFRJdXhWxch7fnzrFgdbeeqjv6o=202).

Reviewer #3: No

Reviewer #5: No
